# Cervical Scrofula Revealing Miliary Tuberculosis and Previously Undiagnosed Advanced HIV: A Case Report

**DOI:** 10.7759/cureus.116443

**Published:** 2026-09-17

**Authors:** Youssef Habib, Jeris Abuhouran, Medin Tafa

**Affiliations:** 1 Medical School, St. George's University, St. George's, GRD; 2 Internal Medicine, Jersey Shore University Medical Center, Neptune City, USA; 3 Internal Medicine, Hackensack Meridian Health Jersey Shore University Medical Center, Neptune, USA

**Keywords:** cervical scrofula, extrapulmonary tuberculosis, human immunodeficiency virus, immune reconstitution inflammatory syndrome, lymphadenopathy, miliary tuberculosis

## Abstract

Cervical tuberculous lymphadenitis, or scrofula, may be the first manifestation of previously undiagnosed advanced HIV, even when concurrent miliary pulmonary tuberculosis produces few or no respiratory symptoms. A 25-year-old male immigrant from Eastern Europe presented with one to two weeks of progressive right cervical and jaw swelling, fever, night sweats, fatigue, and anorexia, without cough, dyspnea, or chest pain. Neck and chest computed tomography demonstrated necrotic bilateral cervical lymphadenopathy, supraclavicular and paratracheal adenopathy, and diffuse miliary pulmonary nodularity. HIV testing revealed advanced untreated infection with a CD4 count of 174 cells/mm³ and HIV-1 RNA of 956,000 copies/mL; QuantiFERON-TB Gold (QIAGEN, Hilden, Germany) was positive. Cervical lymph node biopsy demonstrated necrotizing granulomatous inflammation with acid-fast bacilli. Because the patient could not produce sputum, bronchoscopy with bronchoalveolar lavage (BAL) was performed, and BAL *Mycobacterium tuberculosis* (MTB) polymerase chain reaction detected MTB complex with rifampin resistance not detected. He was started on rifampin, isoniazid, pyrazinamide, and ethambutol therapy with pyridoxine and *Pneumocystis jirovecii* pneumonia prophylaxis. Antiretroviral therapy (ART) with tenofovir disoproxil fumarate/emtricitabine and dolutegravir was initiated six weeks after antituberculous therapy. Two weeks after ART initiation, he developed recurrent neck swelling and fever with new contralateral cervical lymphadenopathy, while HIV-1 RNA had declined to 2,180 copies/mL and CD4 count had risen to 190 cells/mm³. After consideration of treatment failure, drug resistance, bacterial infection, other opportunistic infection, and malignancy, the presentation was felt to be most consistent with paradoxical tuberculosis-associated immune reconstitution inflammatory syndrome (TB-IRIS). Symptoms improved with conservative management and close monitoring. He completed the 60-day intensive phase of antituberculous therapy, transitioned to rifampin and isoniazid continuation therapy, and remained on ART. This case highlights that cervical scrofula may reveal advanced, previously undiagnosed HIV with radiographically extensive but clinically silent miliary tuberculosis, and that recurrent lymphadenopathy after ART initiation should prompt consideration of paradoxical TB-IRIS while alternative diagnoses are evaluated.

## Introduction

Tuberculosis (TB) remains a leading cause of death among people with HIV, and extrapulmonary disease becomes increasingly common as immunosuppression progresses, occurring in roughly 70% of patients with CD4 counts ≤100 cells/mm³ compared with about 30% of those with counts >300 cells/mm³ [1]. Cervical tuberculous lymphadenitis is among the most common extrapulmonary manifestations of TB in people with HIV [2]. Although disseminated TB is well recognized in advanced HIV, less attention has been given to presentations in which striking miliary pulmonary involvement is accompanied by minimal or absent respiratory symptoms and cervical lymphadenopathy serves as the first clinical clue to previously undiagnosed HIV. This radiographic-clinical discordance may delay recognition if clinicians rely on pulmonary symptoms to raise suspicion for TB. We report this case to highlight cervical scrofula as a sentinel presentation of advanced, previously undiagnosed HIV with clinically silent miliary tuberculosis and to emphasize the subsequent risk of paradoxical TB-associated immune reconstitution inflammatory syndrome (TB-IRIS) following antiretroviral therapy (ART) initiation.

## Case presentation

A 25-year-old male immigrant from Eastern Europe with a history of asthma and chronic tonsillitis presented with one to two weeks of progressive right-sided neck and jaw swelling, fever, chills, night sweats, fatigue, and decreased appetite. He denied dyspnea, productive cough, or chest pain. Initial differentials included odontogenic infection, parotitis, sialadenitis, and lymphoma. CT of the chest demonstrated innumerable, diffuse, bilateral, randomly distributed pulmonary micronodules consistent with a miliary pattern, raising concern for disseminated TB (Figure 1). CT of the neck with contrast revealed extensive bilateral cervical lymphadenopathy with cystic and necrotic nodes, greater on the right, as well as supraclavicular and paratracheal adenopathy, consistent with cervical tuberculous lymphadenitis (Figure 2).

**Figure 1:**
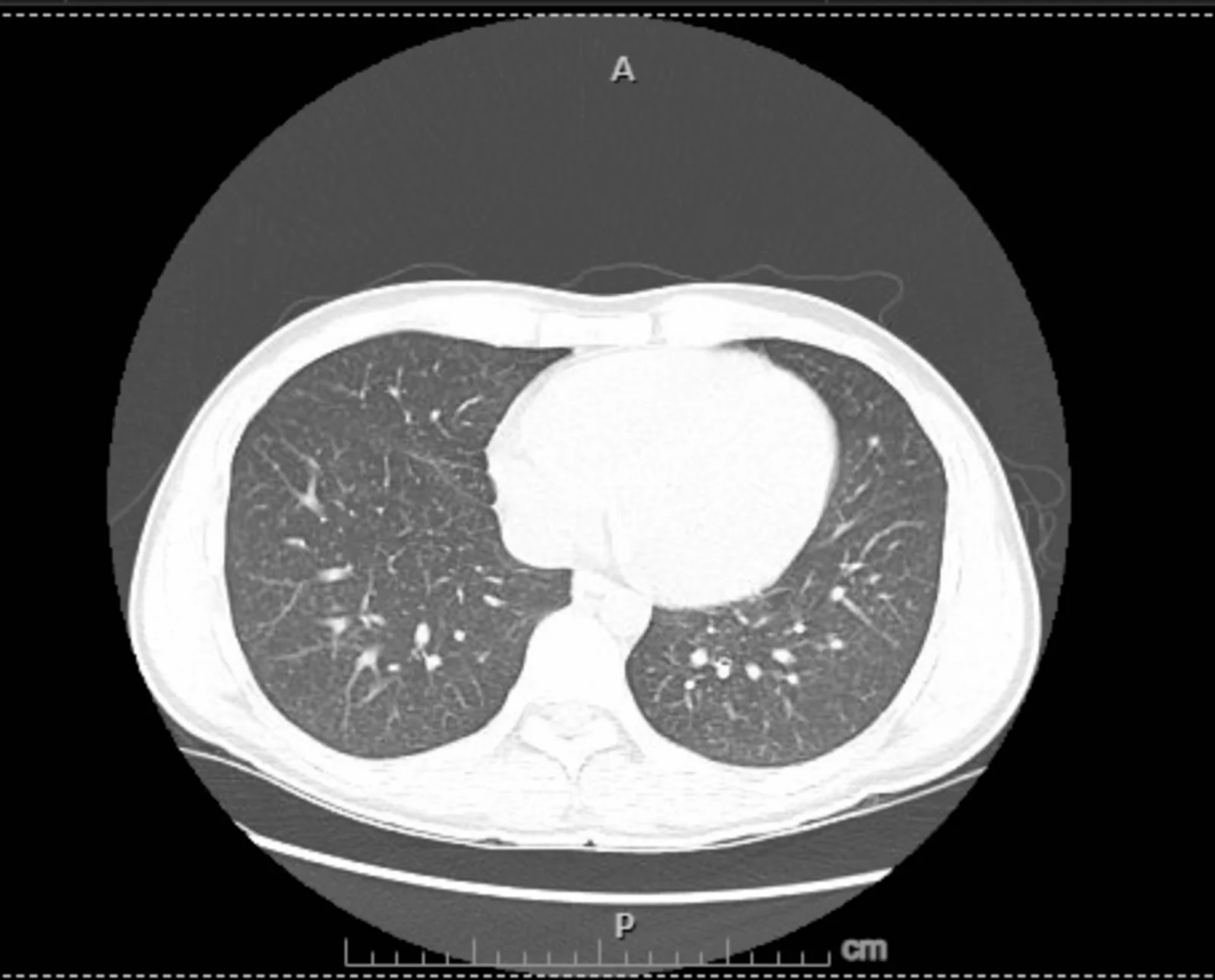
Miliary Pulmonary Tuberculosis CT chest demonstrating diffuse bilateral randomly distributed pulmonary micronodules consistent with a miliary pattern, with representative nodules indicated by arrows.

**Figure 2:**
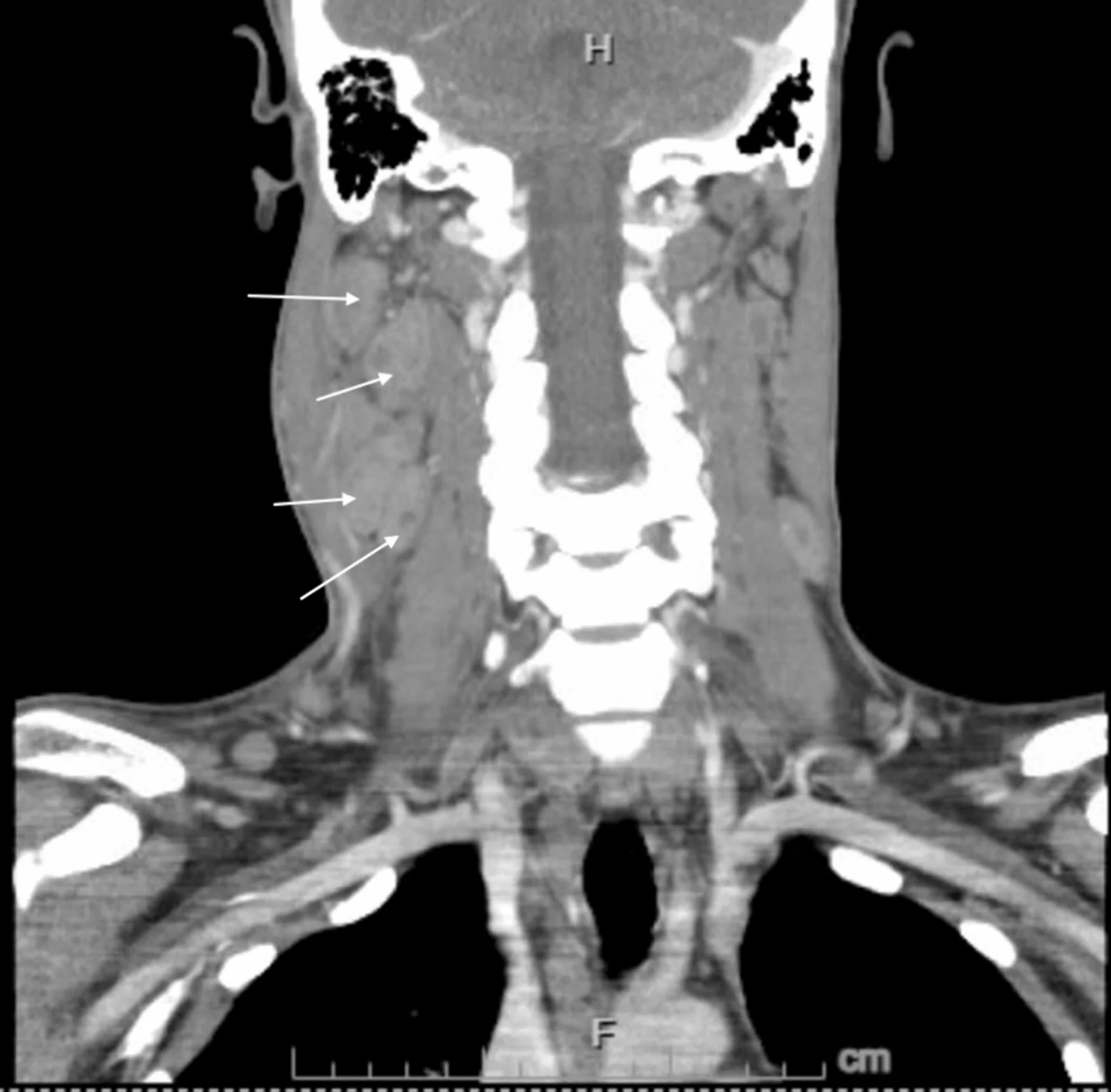
Cervical Tuberculous Lymphadenitis (Scrofula) Coronal contrast-enhanced CT of the neck demonstrating extensive bilateral cervical lymphadenopathy, greater on the right, with central low attenuation consistent with necrotic cervical lymph nodes and cervical tuberculous lymphadenitis/scrofula. Arrows indicate representative necrotic nodes.

Laboratory findings revealed lymphopenia and mild hyponatremia. HIV diagnostic testing was positive by fourth-generation HIV-1/2 antigen/antibody screening, with confirmatory testing consistent with HIV-1 infection. Baseline immune and virologic evaluation demonstrated a CD4 count of 174 cells/mm³ and an HIV-1 RNA level of 956,000 copies/mL, consistent with advanced untreated HIV infection. QuantiFERON-TB Gold (QIAGEN, Hilden, Germany) was positive. Despite extensive radiographic disease, the patient maintained normal oxygen saturation on room air and was unable to produce spontaneous sputum. Interventional radiology-guided core biopsy of a right cervical lymph node was performed to evaluate the necrotic cervical lymphadenopathy. Because a sputum specimen was not available for microbiologic testing, bronchoscopy with bronchoalveolar lavage (BAL) of the right upper lobe was pursued for confirmation. BAL *Mycobacterium tuberculosis* (MTB) polymerase chain reaction confirmed MTB complex, with rifampin resistance not detected (Table 1).

**Table 1:** Key Laboratory and Microbiologic Findings During Hospitalization Abbreviations: A/G, albumin-to-globulin ratio; MTB, *Mycobacterium tuberculosis*; PCR, polymerase chain reaction; BAL, bronchoalveolar lavage; AST, aspartate aminotransferase; ALT, alanine aminotransferase; INR, international normalized ratio; SARS-CoV-2, severe acute respiratory syndrome coronavirus 2; RSV, respiratory syncytial virus

Test	Patient Value	Reference Range	Interpretation
WBC	6.6 ×10³/µL	4.5-11.0 ×10³/µL	Normal
Hemoglobin	13.3 g/dL	13.2-17.5 g/dL	Low-normal
Platelets	204 ×10³/µL	140-450 ×10³/µL	Normal
Absolute lymphocyte count	1.14 ×10³/µL	1.5-3.5 ×10³/µL	Low
Sodium	132 mmol/L	136-145 mmol/L	Low
Total protein	9.5 g/dL	6.0-8.0 g/dL	High
Albumin	3.2 g/dL	3.4-5.0 g/dL	Low
A/G ratio	0.5	>1.0	Low/reversed
AST	44 U/L	0-34 U/L	Mildly elevated
ALT	58 U/L	10–49 U/L	Mildly elevated
INR	1.2	0.9-1.1	Mildly elevated
Procalcitonin	0.14 ng/mL	<0.50 ng/mL	Normal
Lactate	1.9 mmol/L	0.5-<2.0 mmol/L	Normal
HIV Ag/Ab	Reactive	Nonreactive	Positive
HIV-1 confirmatory test	Positive	Negative	Positive
CD4 count	174 cells/mm³	~500-1,500 cells/mm³	Low
QuantiFERON-TB Gold (QIAGEN, Hilden, Germany)	Positive	Negative	Positive
Cervical lymph node biopsy	Necrotizing granulomatous inflammation with rare acid-fast bacilli; tissue MTB PCR, culture, and susceptibility results not available in records reviewed	No granulomas or acid-fast bacilli	Positive for mycobacterial infection; tissue microbiologic confirmation unavailable
MTB PCR	MTB* *complex detected; final BAL culture and full susceptibility results not available in records reviewed	Not detected	Positive
Rifampin resistance	Not detected	Not detected	No rifampin resistance detected
Blood cultures	No growth after 4 days	No growth	Negative
SARS-CoV-2/Influenza/RSV PCR	Negative	Negative	Negative
Rapid Group A Strep	Negative	Negative	Negative
Monospot	Negative	Negative	Negative

The patient was started on rifampin, isoniazid, pyrazinamide, and ethambutol (RIPE) therapy with pyridoxine supplementation and trimethoprim-sulfamethoxazole for *Pneumocystis jirovecii* pneumonia prophylaxis. ART was intentionally deferred, with plans to initiate within two to eight weeks per Department of Health and Human Services (DHHS) guidelines for patients with CD4 counts ≥50 cells/mm³ [1,2]. ART, consisting of tenofovir disoproxil fumarate/emtricitabine and dolutegravir, was initiated six weeks after starting antituberculous therapy, in accordance with DHHS guidelines [1,2]. Because the patient was receiving rifampin-containing antituberculous therapy, dolutegravir was dosed at 50 mg twice daily during rifampin coadministration, consistent with guideline-based recommendations. Trimethoprim-sulfamethoxazole for *Pneumocystis jirovecii* pneumonia prophylaxis was discontinued after a resolving drug rash, and prophylaxis was changed to dapsone 100 mg daily. G6PD testing was performed before dapsone initiation and was within normal limits.

Approximately two weeks after ART initiation, the patient developed recurrent right-sided neck swelling and fever with new contralateral cervical lymphadenopathy, without tenderness, fluctuance, erythema, or drainage, raising concern for paradoxical TB-IRIS. HIV-1 RNA had declined to 2,180 copies/mL and CD4 count had risen to 190 cells/mm³, supporting immune recovery after ART initiation. Given the patient’s recurrent lymphadenopathy and systemic symptoms after ART initiation, the differential diagnosis included paradoxical TB-IRIS, TB treatment failure, drug-resistant TB, bacterial lymphadenitis or superinfection, other opportunistic infections, and malignancy. Malignancy was considered less likely given the initial lymph node biopsy findings, which showed necrotizing granulomatous inflammation without evidence of malignancy. Treatment failure and drug-resistant TB were also considered; however, the patient had prior diagnostic confirmation of TB, was receiving antituberculous therapy, and BAL MTB PCR had shown rifampin resistance not detected. No alternative bacterial or opportunistic infection was identified in the available records. Given the temporal association with ART initiation and the absence of documented evidence supporting these alternative diagnoses, the presentation was felt to be most consistent with a paradoxical TB-IRIS-related inflammatory process. Symptoms were managed conservatively with close monitoring, with corticosteroids reserved should symptoms worsen. The intensive phase of RIPE therapy was completed after 60 days, and the patient transitioned to a four-month continuation phase of rifampin and isoniazid, while ART and antituberculous therapy were continued.

## Discussion

This case illustrates the radiographic-clinical discordance characteristic of TB in advanced HIV. With CD4 counts below 200 cells/mm³, impaired granuloma formation can yield atypical presentations, including miliary patterns and lymphadenopathy rather than classic cavitary disease [3,4]. Patients with low CD4 counts show more lymphadenopathy and miliary patterns but less cavitation than those with higher CD4 counts [3]. Sputum smear microscopy has poor sensitivity in people with HIV, and many hospitalized patients with HIV infection are unable to produce sputum samples [4,5]. Absent respiratory symptoms despite extensive imaging findings may reflect blunted immune-mediated inflammation. Subclinical TB, defined by positive cultures without symptoms, has been reported among people with untreated HIV [4]. Scrofula remains an under-appreciated manifestation of extrapulmonary TB in undiagnosed HIV, and lymph node involvement is common in HIV-related TB [6,7].

This patient carried significant risk for IRIS given his CD4 count, high viral load, and extrapulmonary disease. A meta-analysis reported a pooled paradoxical TB-IRIS incidence of 18% among adults with HIV-associated TB initiating ART, with death attributed to TB-IRIS in 2% of cases [4,8]. Consistently identified risk factors include low CD4 count, high HIV viral load, disseminated or extrapulmonary TB, and a short interval between starting TB treatment and initiating ART [7,9,10]. Common manifestations of paradoxical TB-IRIS include recurrent respiratory symptoms with worsening pulmonary infiltrates and enlarged lymph nodes, which may form pus, resembling this patient's disease pattern [4]. DHHS guidelines recommend ART initiation in HIV-associated TB based on CD4 count, TB site, disease severity, and IRIS risk. For ART-naïve patients with CD4 counts >50 cells/mm³ and no suspected CNS or tuberculous meningitis involvement, ART is generally initiated within two to eight weeks after starting antituberculous therapy [7,11,12]. In this case, ART was initiated six weeks after antituberculous therapy given the patient’s CD4 count of 174 cells/mm³, absence of suspected CNS involvement, disseminated disease, and concern for IRIS risk.

## Conclusions

This case highlights several practical considerations for clinicians. In immunocompromised patients with unexplained cervical lymphadenopathy, particularly when nodes are necrotic, TB should be considered even in the absence of cough or other respiratory symptoms. HIV testing and chest imaging should be considered when TB is suspected. When sputum cannot be obtained, lymph node sampling for histopathology and mycobacterial testing may aid diagnosis, with bronchoscopy considered when additional pulmonary microbiologic confirmation is needed. After ART initiation, new or recurrent fever and lymphadenopathy should prompt evaluation for paradoxical TB-IRIS while also assessing for treatment failure, drug resistance, bacterial or opportunistic infection, and malignancy. Early recognition of these patterns may help reduce diagnostic delay and facilitate coordinated treatment of TB and HIV.
